# Pharmaceutical Drugs and Natural Therapeutic Products for the Treatment of Type 2 Diabetes Mellitus

**DOI:** 10.3390/ph14080806

**Published:** 2021-08-17

**Authors:** Jana Blahova, Monika Martiniakova, Martina Babikova, Veronika Kovacova, Vladimira Mondockova, Radoslav Omelka

**Affiliations:** 1 Department of Botany and Genetics, Faculty of Natural Sciences, Constantine the Philosopher University in Nitra, 949 74 Nitra, Slovakia; jana.blahova@ukf.sk (J.B.); martina.babikova@ukf.sk (M.B.); vmondockova@ukf.sk (V.M.); 2 Department of Zoology and Anthropology, Faculty of Natural Sciences, Constantine the Philosopher University in Nitra, 949 74 Nitra, Slovakia; vkovacova@ukf.sk

**Keywords:** type 2 diabetes mellitus, molecular mechanisms, treatment, pharmaceutical drugs, natural therapeutic products, combination therapy

## Abstract

Type 2 diabetes mellitus (T2DM) is the most widespread form of diabetes, characterized by chronic hyperglycaemia, insulin resistance, and inefficient insulin secretion and action. Primary care in T2DM is pharmacological, using drugs of several groups that include insulin sensitisers (e.g., biguanides, thiazolidinediones), insulin secretagogues (e.g., sulphonylureas, meglinides), alpha-glucosidase inhibitors, and the newest incretin-based therapies and sodium–glucose co-transporter 2 inhibitors. However, their long-term application can cause many harmful side effects, emphasising the importance of the using natural therapeutic products. Natural health substances including non-flavonoid polyphenols (e.g., resveratrol, curcumin, tannins, and lignans), flavonoids (e.g., anthocyanins, epigallocatechin gallate, quercetin, naringin, rutin, and kaempferol), plant fruits, vegetables and other products (e.g., garlic, green tea, blackcurrant, rowanberry, bilberry, strawberry, cornelian cherry, olive oil, sesame oil, and carrot) may be a safer alternative to primary pharmacological therapy. They are recommended as food supplements to prevent and/or ameliorate T2DM-related complications. In the advanced stage of T2DM, the combination therapy of synthetic agents and natural compounds with synergistic interactions makes the treatment more efficient. In this review, both pharmaceutical drugs and selected natural products, as well as combination therapies, are characterized. Mechanisms of their action and possible negative side effects are also provided.

## 1. Introduction

**Figure 1:**
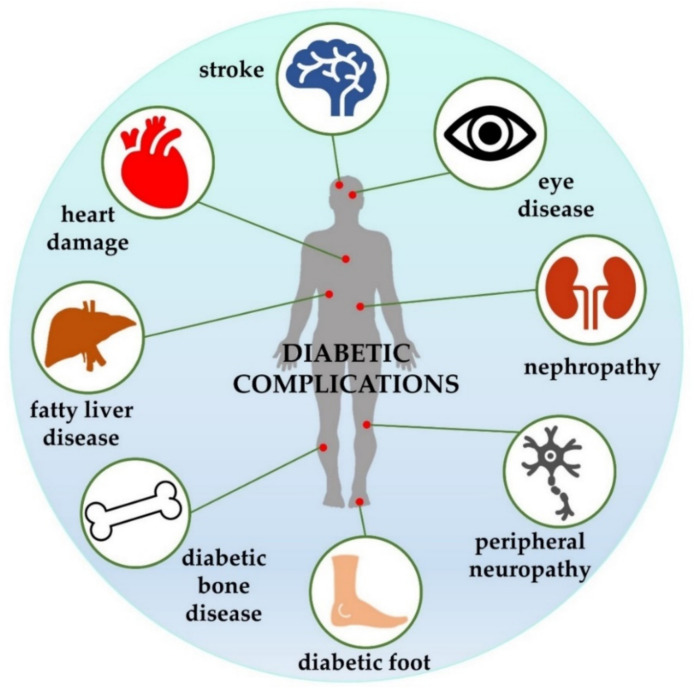
Selected secondary complications associated with T2DM.

Type 2 diabetes mellitus (T2DM) is the most widespread form of diabetes across all continents. According to the World Health Organization, the number of people with this disease will increase almost two-fold in the next 10 years [1]. T2DM is a chronic endocrine disorder characterized by hyperglycaemia, insulin resistance, ineffective insulin secretion by the pancreas [2,3], and increased hepatic glucose production [4]. These conditions manage lower glucose transport to the liver, muscle cells and fat cells [5]. An important feature of diabetes is hyperlipidaemia, which results from the use of lipids instead of glucose [6,7]. Patients with T2DM are at a higher risk of microvascular complications (e.g., diabetic retinopathy, nephropathy, and neuropathy) and macrovascular ones (such as cardiovascular disease, stroke, and peripheral artery disorder) than the non-diabetic population. Other clinical conditions associated with T2DM include diabetic foot and reduced resistance to various infections [8,9,10]. The clinical consequences of hyperglycaemia are polyuria, weight loss (sometimes with polyphagia), and blurred vision [11]. Renal impairment, impaired growth and development, lipodystrophy, non-alcoholic fatty liver damage, limited joint mobility, and oedema can also be diagnosed [10]. In addition, diabetic bone disease is reported as an important secondary complication of T2DM. This abnormality is manifested by altered bone mineral density (BMD), disorders of skeletal microarchitecture and bone metabolism, decreased bone strength, and the lower expression of genes associated with osteoblast function [12,13]. As a result, almost all organ systems can be adversely affected by T2DM-related complications. Selected diabetic complications are illustrated in Figure 1.

The main aim of this review is to examine the available pharmaceutical drugs and selected natural therapeutic products that are commonly used to treat T2DM, as well as to characterize their mechanisms of action and possible negative side effects. Combination therapy comprising of synthetic drugs and natural health compounds is also described. Molecular mechanisms of T2DM are also provided in order to better understand this issue.

## 2. Molecular Mechanisms of Type 2 Diabetes Mellitus

**Figure 2:**
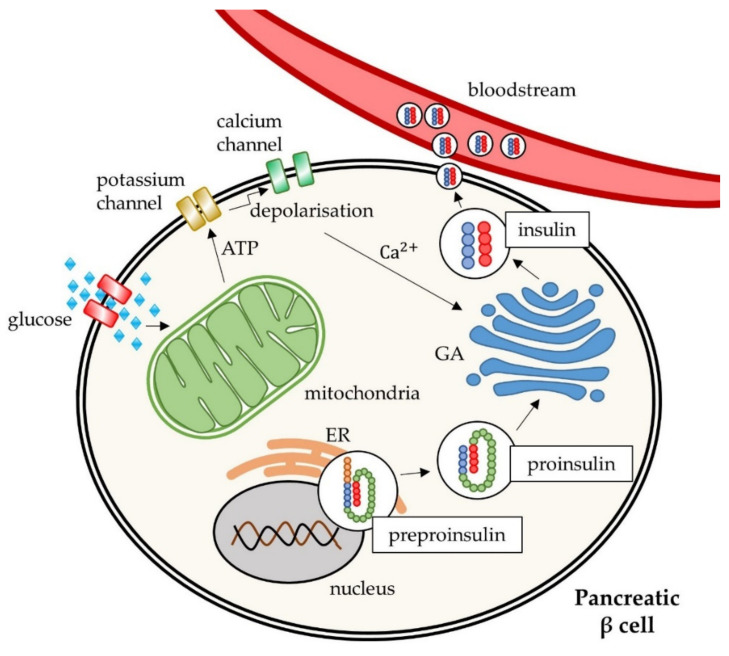
The mechanism of insulin release by pancreatic β-cells. ER: endoplasmic reticulum, GA: Golgi apparatus, ATP: adenosine triphosphate, Ca^2+^: calcium ion.

At the molecular level, T2DM is manifested by two major pathological defects: impaired insulin secretion through pancreatic β-cells dysfunction, and defective insulin action due to insulin receptor abnormalities [10,14]. Impaired β-cell function leads to a decrease in glucose responsiveness [15] and its insufficient treatment reduces the number of pancreatic β-cells and affects long-term blood glucose control [16]. Insulin doses are high during chronic hyperglycaemia and T2DM, leading to the exhaustion of β-cells and deficiency in insulin biosynthesis and secretion [17]. Generally, insulin synthesis begins as preproinsulin production through kinase-dependent signalling pathways (PI3K, p38MAPK, PKA, calmodulin kinase). Preproinsulin is then inserted into the endoplasmic reticulum, where it is metabolized to proinsulin. Subsequently, proinsulin is transported to the Golgi apparatus, where insulin is formed [18]. The main stimulator of insulin secretion is elevated blood glucose level. Glucose enters the pancreatic β-cells via a low-affinity glucose transporter (GLUT-2 in rodents, and GLUT-1 and GLUT-3 in humans), which is then phosphorylated. Pyruvate is then generated by glycolysis in the cytoplasm and metabolized by pyruvate carboxylase and pyruvate dehydrogenase in mitochondria. This process leads to an increase in the cytoplasmic adenosine triphosphate (ATP)/adenosine diphosphate (ADP) ratio, resulting in the closure of ATP-sensitive K^+^ channels. The closure of these channels is associated with another process that leads to activation of L-type voltage-dependent calcium (Ca^2+^) channels and ultimately to the release of insulin into the circulation [19]. Figure 2 shows the mechanism of insulin release by β-cells. The regulation of insulin secretion is controlled by nutrients (e.g., glucose, free fatty acids), endocrine peptides, hormones (e.g., insulin, leptin, incretins, growth hormone, prolactin, glucagon), and the autonomic nervous system [20]. The molecular background of diabetic pancreatic β-cell dysfunction is based on cellular stress in the endoplasmic reticulum and mitochondrial dysfunction. These conditions are influenced by hyperglycaemia (glucotoxicity), hyperlipidaemia (lipotoxicity), and chronic inflammation. Inflammation is characterized by an imbalance between compensatory mechanisms and elevated levels of pro-inflammatory cytokines and chemokines [17,21]. In the diabetic state, a defect in hepatic glucose production is recorded. Insulin action is generally reduced in the liver and peripheral tissues and causes elevated hepatic gluconeogenesis and glycogenolysis, decreased glucose uptake by peripheral tissues, and higher glucose levels in the blood [22].

Insulin resistance, typical for T2DM, is a condition in which the blood concentration of insulin does not exert its biological activity [16,23]. Factors that affect insulin resistance include age, exercise, diet, and body fat distribution [24]. It occurs in peripheral tissues (especially in adipose, hepatic, and muscular tissues), and results in an increased amount of secreted insulin [10,25]. Generally, insulin receptors are part of the receptor family with tyrosine kinase activity. After insulin binds to its receptor, there are conformational changes of the receptor and autophosphorylation, and the activation of receptor’s tyrosine kinases is observed. These changes lead to the tyrosine phosphorylation of insulin receptor substrates (IRSs), including IRS1-4, growth factor receptor-binding protein 2-associated binder-1 (Gab1), and Shc. IRSs are considered to be adapter molecules which organize the formation of molecular complexes and start cascades of intracellular signalling [25,26]. In general, insulin has two signalling pathways. The first is consistent with the activation of the phosphatidylinositol-3-kinase (PI3K)/Akt pathway (also known as protein kinase B, PKB). PI3-K stimulation promotes increased nitric oxide (NO), Na^+^ pump, K^+^ channel, and Ca^2+^ myofilament sensitivity. This pathway activates insulin or insulin-like growth factor-1 (IGF-1). The second pathway acts through the mitogen-activated protein kinases/Ras pathway regulating gene expression and insulin-associated mitogenic effects [14,25]. In patients with T2DM, impaired insulin activation is the result of defects in the IRS-1/PI3K/Akt signalling pathway. These conditions have detrimental effects on insulin action and glucose metabolism. These defects may be partially resolved by treatment with insulin-sensitising agents and effective lifestyle changes [26].

## 3. Treatment of Type 2 Diabetes Mellitus

The recommended initial approach to T2DM management involves a combination of efficient lifestyle changes and the use of pharmaceutical drugs. It is a way to achieve good metabolic control. The two main lifestyle changes include a healthy diet and physical activity [27,28]. Supposedly, physical activity can reduce the risk of T2DM development by 30–50%, due to a lower incidence of obesity [3]. Physical activity contributes to enhanced insulin sensitivity in tissues, improves glucose and lipid levels, regulates blood pressure, and supports the cardiovascular system, which together lead to weight loss and a better quality of life [28]. Caloric intake and diet quality are also important in the management of T2DM. Dietary aspects can also influence blood glucose and lipid concentrations, blood pressure, body weight, and health conditions. Patients with T2DM should avoid smoking, alcohol consumption and limit sodium intake [3]. It is strongly recommended that patients consume unsaturated fats and dietary meat with a low content of saturated and trans fats, foods with a low glycaemic index, sweeteners with a lower caloric value, fresh fruits, and vegetables. A high-fibre diet reduces the risk of cardiovascular diseases. The consumption of products rich in omega-3 fatty acids also reduces this risk [2,28,29]. Furthermore, the addition of minerals, especially chromium (Cr), magnesium (Mg), and vanadium (V) is very important. Chromium is a secondary messenger/cofactor of insulin that increases insulin sensitivity and the activity required for normal glucose and lipid metabolism. Magnesium is a mediator of insulin action and vanadium has insulin-like effects on the metabolism of glucose, enhances insulin action, and the sensitivity of its receptors [30,31,32]. T2DM is strongly associated with oxidative stress, which is consistent with an increased prevalence of secondary diabetic complications. Various damages caused by oxidative stress can be limited by vitamins with antioxidant properties (e.g., vitamins C, E and A) [29,33]. Functional foods, for example whole-grain diet biscuits, whole-grain breakfast cereals, and bee products, can also eliminate diabetic problems [34]. A higher intake of whole grains (unlike refined grain) lower the risk of T2DM mainly due to their high fibre content. Dietary fibres decrease starch accessibility to alpha-amylase and reduce glucose diffusion; stimulate satiety signals; and affect production of hormones involved in body weight regulation and energy homeostasis, as well as in glucose control [35]. Other bioactive compounds such as phenolic compounds, phytosterols, betaine, and carotenoids may also contribute to an improved insulin sensitivity by hampering oxidative stress, the transcription of inflammatory cytokines and chronic low-grade inflammation. In addition, vitamins (e.g., B and E) and minerals (e.g., Mg, zinc (Zn), and selenium (Se)) found in whole grains can reduce oxidative stress and inflammation [36]. Capcarova et al. [37] and Martiniakova et al. [38] state the protective impact of bee bread against hyperglycaemia, hyperlipidaemia, and diabetic bone disease in the appropriate T2DM animal model. Moreover, the most famous bee product, honey, can also improve glycaemic control and reduce secondary diabetic complications [39]. Honey is composed mostly of saccharides (mainly fructose) and water, but it also contains a wide range of nutrients including proteins, fatty acids, prebiotics, probiotics, fibre, phytochemicals, bioactive peptides, minerals (e.g., Se, Zn, copper (Cu), and V), vitamins, organic acids, phenolic acids, flavonoids, and carotenoids [34]. Its hypoglycaemic properties are attributed to several mechanisms, the most important mechanism is based on fructose action. Fructose has lower glycaemic index when compared to glucose. Its mechanisms of action may include a reduced rate of intestinal absorption, the prolongation of gastric emptying time, and a reduced food intake. Fructose stimulates glucokinase and inhibits phosphorylase activity in the liver. The aforementioned minerals, phenolic acids, and flavonoids might also have a role in the hypoglycaemic process. In addition, honey has antioxidant properties and reduces oxidative stress in pancreatic β-cells [40].

The possibilities of the pharmacological therapy of T2DM are focused on its main pathophysiological defects (insulin resistance and insufficient insulin secretion) and subsequent diabetic complications [41,42]. However, the long-term application of pharmacological agents can be associated with many harmful side effects, emphasising the importance of natural therapeutic compounds. The acceptance and use of natural health products as alternative therapies is dramatically increasing because natural product-derived drugs are more affordable, with fewer side effects compared to conventional pharmaceutical therapies. In addition, the combination therapy of synthetic drugs and natural compounds has the potential to make the treatment more effective, especially in the advanced stage of T2DM [43]. Here, we introduce some pharmaceutical options, natural therapeutic products, selected plant fruits, vegetables, and other products useful in the treatment of T2DM.

### 3.1. Pharmaceutical Drugs for the Treatment of Type 2 Diabetes Mellitus

The first pharmacological treatment of T2DM came from insulin isolated from an animal pancreas in the 1920s. In recent years, many orally administered agents and injectable drugs have been developed to treat patients with T2DM. They can be used individually and/or in combination. These medications are aimed at enhancing blood glucose levels, and reducing body weight and the risk of cardiovascular damage [41,44]. However, the efficacy of drugs for weight loss varies and can range from mild weight loss (less than 3.2% of the initial weight) to strong weight loss (greater than 5% of the initial weight) [45]. Besides, none of these synthetic drugs are free from adverse effects. These include hypoglycaemia, lactic acidosis, gastrointestinal disorders, weight gain, renal failure, and diarrhoea, etc. According to Raptis and Dimitriadis [46], Drucker et al. [47] and Nathan et al. [48], antidiabetic drugs can be divided into the following categories: insulin sensitisers (e.g., biguanides and thiazolidinediones), insulin secretagogues (e.g., sulphonylureas and meglitinides), alpha-glucosidase inhibitors and incretin-based therapies (e.g., glucagon-like peptide-1 receptor agonists and dipeptyl peptidase-4 inhibitors). Other groups represent the sodium-glucose cotransporter 2 inhibitors (decreased hyperglycaemia by enhanced urinary glucose excretion) [49], lipase inhibitors [46], amylin agonists (analogues of the β-cell hormone amylin), and insulin [48].

#### 3.1.1. Insulin Sensitisers

Insulin sensitisers have the ability to decrease insulin resistance in T2DM. The low insulin response (resulting in insulin resistance) represents an early defect in the T2DM period that occurs years before the onset of hyperglycaemia and the clinical formation of diabetes. Insulin sensitisers not only improve insulin sensitivity, but also positively influence metabolic abnormalities associated with T2DM, such as impaired lipid metabolism and harmful atherosclerotic vascular processes [50]. Pharmaceutical drugs that improve insulin sensitivity include biguanides and thiazolidinediones.

##### Biguanides

Biguanides are among the most commonly recommended oral antihyperglycaemic pharmaceutical agents. The major biguanide used to treat non-insulin-dependent T2DM is metformin (dimethyl biguanide) [51,52]. The source of metformin is galegine, a natural product produced by the plant Galega officinalis [53,54]. The impact of metformin is associated with decreased hepatic glucose production, increased insulin sensitivity in peripheral tissues, and reduced insulin levels during fasting. The downregulation of hepatic glucose production occurs via AMP-activated protein kinase (AMPK), which promotes mitochondrial activity in cells. The mitochondrial control of hepatic gluconeogenesis leads to lower cellular energy and gluconeogenesis. Supposedly, insulin resistance may be improved through the beneficial effects of metformin on the expression of the insulin receptor and the activity of tyrosine kinase. Another mechanism includes an enhanced expression of the incretin receptor gene through the action of glucagon-like peptide 1, leading to the stimulation of β-cells [22,54,55]. Moreover, metformin positively affects diabetic bone structure through increased osteoblast proliferation, type I collagen production and alkaline phosphatase activity [56,57]. Metformin treatment can be consistent with the elevated possibility of lactic acidosis (characterized by excessively low pH in the bloodstream), as well as with gastrointestinal adverse effects (diarrhoea and nausea being the most common) [58,59,60].

##### Thiazolidinediones

The group of thiazolidinediones, also referred to as glitazones or peroxisome proliferator-activated receptor-γ (PPAR-γ) agonists, involves pioglitazone and rosiglitazone [61,62]. The application of thiazolidinediones leads to a reduction of blood glucose levels, an improvement in β-cells function [61], and a decrease in insulin resistance (especially in adipose and liver tissues) [63]. Their application is also associated with lower levels of circulating free fatty acids [61], anti-inflammatory effects [64], and reduced risk of cardiovascular diseases [65]. These improvements are caused by the activation of the nuclear transcription factor PPARγ [63], which is primarily found in adipose tissue, and is involved in glucose, lipid and protein metabolisms. Generally, thiazolidinediones have a high affinity to the PPARγ receptor [66]. The PPARγ agonists increase the expression and secretion of the hormone adiponectin in adipocytes which suppresses insulin resistance [67]. Nonetheless, PPARγ rceptors are expressed on bone-marrow-pluripotent mesenchymal stem cells differentiating into adipocytes or osteoblasts, and their stimulation may lead to improved adipocyte differentiation instead of osteoblast differentiation. PPARγ stimulation also supports osteoclast development. As a result, bone loss is significant after one year of treatment with a higher risk of fractures. These conditions are manifested by a reduced osteoblast number, decreased blood vessel formation, and increased number of adipocytes at the healing site [57,68]. Furthermore, another well-known adverse impact is weight gain. Supposedly, weight gain may be caused by disturbances in adipose tissue distribution. Other side effects include fluid retention, oedema, anaemia, and the risk of cardiac failure. In patients treated with these medications, upper respiratory tract infections, sinusitis, and headaches can also be diagnosed [61,64].

#### 3.1.2. Insulin Secretagogues

Insulin secretagogues may support the first phase of insulin secretion (specific by the rapid onset of insulin) and can stimulate accelerated short-term insulin release. On the other hand, their effect on the second phase of insulin action (specific by slower insulin release and lasting until hyperglycaemia) is not permanent [68,69,70]. The first phase of the insulin response is reduced and is partly responsible for the postprandial adjustment of plasma glucose in T2DM. After a prolonged duration of the disease, the second phase of the insulin response is reduced [69]. Insulin secretagogues include sulphonylureas and meglitinides.

##### Sulphonylureas

Sulphonylureas are derivates of sulphonamide with an affinity to the pancreatic β-cell sulphonylurea receptor. They stimulate insulin release through a direct action on β-cells independently of glucose. However, they probably do not produce insulin from the dysfunctional pancreas in the latest stage of T2DM [71,72]. Sulphonylureas are generally divided into three generations: the first includes tolbutamide, tolazamide, chlorpropamide, and acetohexamide; the second consists of glyburide (also known as glibenclamide), glipizide, and gliclazide; and the third generation consists of glimepiride. These generations have the same mechanism of action; however, the second and third generations greatly support the loss of weight [72,73]. The mechanism of sulphonylureas action relies on their binding to the transmembrane sulphonylurea receptor (SUR-1), a regulatory subunit of ATP-sensitive K^+^ channels, and mediates their closing leading to a release of pre-formed insulin. The main side effect of this process includes hypoglycaemia [68]. The risk of hypoglycaemia is higher in elderly people, and people with multiple medications [74], impaired renal functions [75], and liver disease. Other side effects include weight gain (1–4 kg over 6 months), skin reactions, acute porphyria and, rarely, hyponatraemia [68]. The treatment with sulphonylureas can be critical for patients with cardiovascular disease [76]. Hypoglycaemia is also associated with increased fracture development. On the contrary, sulphonylureas appear to have a beneficial or neutral impact on diabetic bone disorder [77].

##### Meglitinides

Meglitinides, also known as glinides, are synthetic antidiabetic agents [78]. Nateglinide, repaglinide, and mitiglinide are members of this group [79]. They have a different chemical structure: nateglinide is an amino acid derivate, repaglinide is a carbamoyl methyl benzoic acid derivative, and mitiglinide is a derivative of benzylsuccinic acid propionate dihydrate [80]. Glinides have a fast onset but a short duration of action. As sulphonylureas, they bind to the pancreatic β-cell receptor and regulate the closure of K^+^ channels in these cells [78,81]. However, in contrast with sulphonylureas, their association to the sulphonylurea receptor 1 binding site is weaker and their dissociation from this receptor is faster [82]. Glinides are also called “short-acting type insulin secretagogues”, and they primarily control postprandial blood glucose level [79] by increasing insulin secretion [78]. Their application may likely lead to elevated glucagon secretion [83]. Treatment with meglitinides is consistent with weight gain similar to sulphonylureas therapy. The advantage of these medications (in contrast to sulphonylureas) is the decreased percentage of hypoglycaemia events thanks to their weaker binding strength [81,84]. These drugs may also be critical in patients with cardiovascular disease, although their potential side effects are minor due to their shorter half-lives [83]. The other adverse impacts involve rhinitis and bronchitis [80]. No beneficial or negative effects of meglitinides application have been reported on diabetic bone structure so far [85].

#### 3.1.3. Alpha-Glucosidase Inhibitors

Alpha-glucosidase inhibitors (e.g., acarbose, miglitol, and voglibose) have been found as an alternative treatment for T2DM. They may be extracted from medicinal plants or microbes (including bacteria and fungi), or chemically synthesised [86,87]. Alpha-glucosidase is a membrane-bound gastrointestinal hydrolysing enzyme that stimulates the decay of disaccharides and oligosaccharides, and the absorption of glucose monomers in the small intestine [88]. Alpha-glucosidase inhibitors have a high affinity for alpha-glucosidases. Due to their nitrogen content, they are able to block enzymatic reactions [89]. They reduce the upper alpha-glucosidases, leading to a delay in the absorption of carbohydrates in the small intestine. As a result, postprandial blood glucose and insulin levels are reduced [90,91]. Lower postprandial glucose concentration is associated with improved β-cells function. Additionally, alpha-glucosidase inhibitors stimulate GLP-1 release from the gut [85]. They influence body weight gain, support insulin sensitivity, adjust blood pressure, protect against heart disease, and reduce hypertriglyceridaemia. On the contrary, they do not affect diabetic bone disorder. Some undesirable impacts on intestinal disturbances such as vomiting, flatulence, diarrhoea, headache, and insomnia, have been described [85,86,89,92].

#### 3.1.4. Incretin-Based Therapies

Incretins (glucagon-like peptide-1 (GLP-1) and glucose-dependent insulinotropic peptide (GIP)) are proglucagon hormones produced by cells of the small intestine. They stimulate pancreatic β-cells to insulin release by binding to G-protein-coupled receptors highly expressed on islet β-cells and/or via the closing of ATP-sensitive K^+^ channels. They also stimulate β-cells proliferation and neogenesis, and reduce their apoptosis through several mechanisms. In addition, they inhibit the glucagon secretion from pancreatic α-cells. This suppression depends on plasma glucose level. The receptors of GLP-1 and GIP exert indirect metabolic actions because they are widely expressed in different cells and tissues [93,94,95]. It seems that incretin-based antidiabetic drugs have beneficial effects on diabetic bone structure. They inhibit bone resorption and have anabolic properties [77]. The action of incretins can be influenced by several nutrients, mainly glucose and carbohydrates [95]. Their concentrations are small during fasting, but they increase rapidly after food ingestion. From understanding the mechanism of incretin system action, a new class of drugs has been found—incretin mimetics. Current incretin mimetics medications involve GLP-1 agonists and dipeptidyl peptidase-4 (DPP-4) inhibitors with an ability to potentiate incretin receptor signalling [47,96].

##### Glucagon-like Peptide-1 Receptor Agonists

GLP-1 receptor (GLP-1R) agonists are one of the most recently used drugs influencing the endogenous incretin hormone, GLP-1 [97]. GLP-1R is synthesised in the pancreatic islets, kidney, lungs, heart, and nervous system. After the binding of GLP-1 and GLP1-agonists to the receptor, glucose-dependent insulin secretion is induced [94]. GLP-1R agonists, structurally related to the native gut peptide, are administered by a subcutaneous injection. This group consists of exendin-4, liraglutide, exenatide [47], lixisenatide, dulaglutide, albiglutide, and semaglutide [98]. These medications are also able to inhibit glucagon secretion and hepatic glucose production, influence α- and β-cell dysfunction, delay gastric emptying, and induce satiety [94,99]. The decrease of blood glucose, induced by GLP-1 receptor agonists, can be affected by baseline glucose level. For example, in the study of Berra et al. [100], patients with high glycated haemoglobin (HbA1c) at baseline saw its greatest reduction after 6 months of once-a-week administration of dulaglutide. The low baseline of HbA1c and the short duration of diabetes were important predictors of the achievement of HbA1c ≤ 7.0%. The percentage of subjects with HbA1c ≤ 7.0% increased from 7.2% at baseline to 52.7% after 6 months in this study. According to the recent update of guideline ESC-EASD [101], treatment with GLP-1 receptor agonists is recommended as the first option in diabetic patients, particularly in those at risk of cardiovascular disease, because of an important effect on both primary and secondary prevention of ischemic disease. On the other hand, GLP-1 receptor agonists can cause transient gastrointestinal problems (e.g., nausea and diarrhoea), local irritation, local nodule formation (caused by the injection), and pancreatitis [102], and their use further poses a risk to patients with renal impairment or end-stage renal disease [97].

##### Dipeptidyl Peptidase-4 Inhibitors

Dipeptidyl peptidase-4 inhibitors are orally administered incretin mimetics medications. Sitagliptin, vildagliptin, saxagliptin, alogliptin, and linagliptin belong to the family of DPP-4 inhibitors. They are chemically synthesised, but also occur in natural resources, as protease inhibitors (e.g., alkaloids, flavonoids, glycosides, phenolic acids, polysaccharides, peptidoglycans, glycopeptides, and steroids). They downregulate DPP-4, responsible for degradation of GLP-1, and thereby restore GLP-1 to physiological levels [103,104,105]. DPP-4 inhibitors are glucose-lowering agents that influence glucose-dependent insulin secretion, cause a delay in gastric emptying, increase levels of active GLP-1, decrease levels of postprandial glucagon, and reduce food intake [105,106,107]. They mimic many of the actions of GLP-1, including the preservation of β-cell mass [108]. Their other benefits incorporate anti-inflammatory, cardiovascular protective, and potential immunomodulatory effects [104]. They do not cause weight gain, hypoglycaemia, and only a few gastrointestinal side effects have been reported. However, their long-term administration may lead to a risk of new heart failure and/or abnormalities in cardiac remodelling in patients with established heart failure [103,109].

#### 3.1.5. SGLT2 Inhibitors

Inhibitors of the sodium–glucose cotransporter 2 (SGLT2) are a group of blood glucose lowering drugs that block renal glucose reabsorption [110]. In general, all filtered glucose undergoes reabsorption in the kidney tubules and more than 90% of glucose is reabsorbed by SGLT2. Under hyperglycaemic conditions, SGLT2 becomes overexpressed leading to increased reabsorption. Inhibitors of SGLT2 block the reabsorption process, resulting in an increase in urinary glucose excretion [111]. Currently used SGLT2 inhibitors are analogues of phlorizin, a natural compound isolated from the bark of apple trees [112]. Four phlorizin-based SGLT2 inhibitors are commercially available in the U.S. and Europe [45,111]: canagliflozin, dapagliflozin, empagliflozin, and ertugliflozin. In addition, tofogliflozin and luseogliflozin have been approved in Japan. These drugs not only control blood glucose levels but also have the potential to improve cardiovascular system function, reduce kidney disorders, and normalize the lipid profile. They could be used either as monotherapy or in combination with other antidiabetic agents (especially with metformin) in T2DM patients [113]. However, some important side effects have been reported with phlorizin-derived SGLT2 inhibitors, including urinary and genital infections, ketoacidosis, and even an increased risk of bladder cancer (dapagliflozin), or amputation and bone fractures (canagliflozin) [111].

### 3.2. Natural Therapeutic Products for the Treatment of Type 2 Diabetes Mellitus

**Figure 3:**
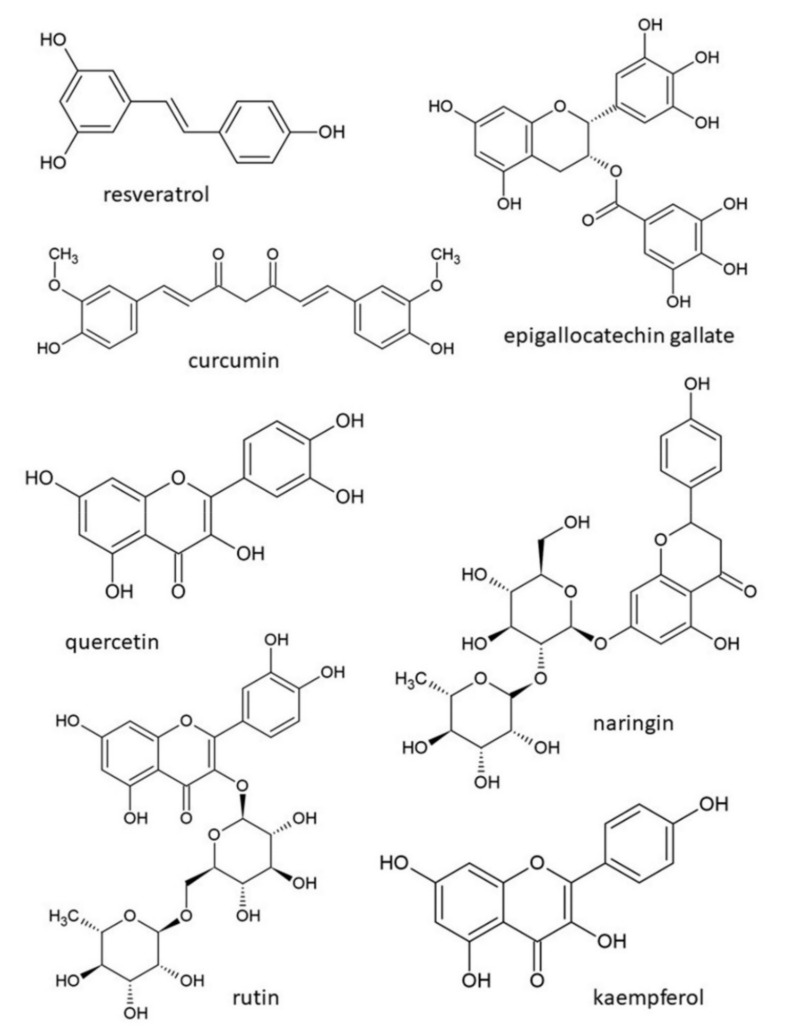
Chemical structures of selected natural compounds.

Active substances of animal origin or substances extracted from plants can be useful in both preventing or supporting the therapy of some pathological conditions, including diabetes mellitus [114]. Currently, there are constant changes in food production and consumption [34]. Natural health products are defined as vitamins, minerals, herbs, spices, and homeopathic and traditional medicines (e.g., Chinese medicines, probiotics, and other products like amino acids and essential fatty acids) [115,116]. Herbal medicine belongs to the category of alternative medicine. The possibility of improving glycaemic control without pharmaceutical drugs or insulin injections is attractive to humans. Natural therapeutic products for the treatment of T2DM focus on main pathophysiological mechanisms, identical to those of pharmaceutical agents, e.g., regulation of insulin secretion, enhancement of insulin resistance, and insulin sensitivity [117,118]. They also influence adverse concentrations of blood lipids, focusing on achieving the suitable energy to balance weight and diabetic complications, and improve health conditions [119]. While the prolonged administration of pharmaceutical drugs may cause many side effects, herbal medicines are non-toxic with a mild action and a minimal risk of contraindications or harmful impacts [120,121]. Some side effects are still unknown due to undiscovered metabolites that are the result of microbial degradation in the human body [122]. Studies and experiments are usually short-term and involve a small sample size. At the present moment, our knowledge of the efficacy and safety of natural health products is lower than that of pharmaceutical products [116]. Another disadvantage of the use of natural compounds is that they are less effective [115], so they are recommended in the pre-diabetic stage, in the early stage of T2DM and in combination with pharmaceutical drugs [123]. In this review, natural health substances are divided into following categories: non-flavonoid polyphenols (e.g., resveratrol, curcumin, tannins, and lignans), flavonoids (e.g., anthocyanins, epigallocatechin gallate, quercetin, naringin, rutin, and kaempferol), and plant fruits, vegetables and other products (e.g., garlic, green tea, blackcurrant, rowanberry, bilberry, strawberry, Cornelian cherry, olive oil, sesame oil, and carrot). Although flavonoids belong to polyphenols, we list them as a separate category due to their high importance in the treatment of T2DM. Figure 3 illustrates chemical structures of selected natural compounds. Their known effects and mechanisms of action are listed in Table 1.

#### 3.2.1. Polyphenols

Polyphenols are polyhydroxyphenols defined as secondary metabolites of plants used for protection against ultraviolet radiation or the aggression of pathogens in plants. They are responsible for the bitterness, astringency, colour, flavour, odour, and oxidative stability of plants. They are often considered natural phytochemicals. Polyphenols can be found in fruits, vegetables, and in products made from them, e.g., cereals and beverages [167]. Tijjani et al. [168] presented the following classes of polyphenols: phenolics, stilbenes, flavonoids, tannins, and lignans. They affect some metabolic processes of the cell, e.g., blocking cell apoptosis and reducing enzymes such as lipoxygenase and telomerase [169]. Their consumption can contribute to the enhanced protection against various diseases such as cancer, cardiovascular disorder, osteoporosis, and neurodegenerative damage [167]. T2DM is one of the most studied diseases that can be affected by the activity of polyphenols [129]. From the group of non-flavonoid polyphenols, resveratrol, curcumin, tannins, lignans with important biological activities are characterized below.

##### Resveratrol

Resveratrol (trans-3,5,4-trihydroxystilbene) is a phytoalexin [125], a plant-derived stilbene with anti-inflammatory, anti-carcinogenic, cartilage-protective, anti-ageing properties, and with the ability to support endothelial cell function. It can improve insulin sensitivity in patients with T2DM, diet-induced obese mice and rats, and Zucker diabetic fatty (ZDF) rats [129,130]. Resveratrol activates deacetylases sirtuins 1–7, mainly a prolific, highly conserved NAD+-dependent lysine deacylase SIRT1. SIRT1 is a potential pharmacological agent targeting insulin resistance in T2DM. In addition, resveratrol has antioxidant properties and can protect β-cells from oxidative stress. It also reduces the risk of diabetic neuropathy [125,126] and interacts with the PPARγ receptor [127]. Another beneficial effect of resveratrol is the reduction of hyperlipidaemia and dyslipidaemia [126,170]. Bo et al. [171] reveal its ability to prevent bone loss in patients with T2DM. Grapes, berries, and peanuts are important sources of resveratrol [125].

##### Curcumin

Curcumin is a yellow component of the turmeric (*Curcuma longa*) plant presented in the rhizome. It has known antioxidant, anti-inflammatory, antimicrobial, immunomodulatory, antitumor, hypoglycaemic, and anti-rheumatic effects. It also has a positive impact on the cardiovascular, renal, and hepatic systems. Another beneficial effect is a reduction in total cholesterol, blood pressure, and platelet aggregation [172,173]. Curcumin decreases postprandial glycaemia and improves insulin sensitivity [128]. The mechanism of curcumin action is possible via the inhibition of lipid peroxidation, NF-κB activation, and the reduction of inflammatory cytokine levels. These conditions support pancreatic cell viability [129,174]. Curcumin is also able to inhibit the activity of alpha-amylase and alpha-glucosidase leading to the delayed absorption of carbohydrates or nutrients in the small intestine [43]. Moreover, a study by He et al. [175] revealed its potential use for bone regeneration at high glucose concentrations. On the other hand, this natural supplement has poor bioavailability [173].

##### Tannins

Tannins are polyphenolic compounds synthesized as secondary metabolites in plants [176]. They can be divided into hydrolyzable and condensed tannins. Condensed tannins are also called proanthocyanidins. Tannins can provide protection against degenerative diseases. They have high antioxidant [177], anti-inflammatory [132], cardiovascular [178], hypoglycaemic [179,180,181], and anti-hypercholesterolemic impacts [182]. They are able to reduce postprandial hyperglycaemia and prevent or delay glucose absorption by inhibiting alpha-glucosidase activities. Other useful characteristics include adipocyte inhibition, an insulin-like effect, glucose transport stimulation, insulin receptor phosphorylation, and GLUT 4 translocation [130,131]. In addition, they reduce the formation and accumulation of AGEs, which are responsible for the development of chronic diabetic complications [132]. These polyphenolic compounds can also prevent cardiovascular damage [182]. The sources of tannins are cereals, legumes, nuts, grapes, apricots, barley, peaches, dried fruits, mint, basil, rosemary, pomegranate, strawberries, rice, and oats. They can also be found in beverages such as green tea, black tea, wine, fruit juice, beer, and coffee [132,176].

##### Lignans

Lignans are polyphenolic compounds found in plants which demonstrate phytoestrogenic activity [183,184]. Some important lignans with bioactive properties are secoisolariciresinol diglucoside (SDG), artigenin, enterodiol, enterolactone, sesamin, syringaresinol, medioresinol, matairesinol, lariciresinol, and pinoresinol. Above all, SDG is the major lignan found mainly in flaxseed. Flaxseed consumption has shown to decrease blood glucose and lipid levels, delay postprandial glucose absorption, and reduce inflammation and oxidative stress in patients with pre-diabetes [133]. In obese mice, flaxseed SDG also lowers blood glucose, insulin, free fatty acids, and improves oral glucose tolerance and insulin signalling [134]. According to Draganescu et al. [136] and Prasad [135], both flaxseed extract and SDG isolated from flaxseed delay the development of T2DM in diabetic rats. Lignans can mediate their effects by a variety of mechanisms, including the modulation of pancreatic insulin and alpha-amylase secretion, increasing insulin sensitivity, and improving their antioxidative capacity. Wang et al. [134] demonstrated the ability of flaxseed SDG to increase the expression of GLUT4 proteins in the muscle tissue. Lignans may also act via estrogen receptor-mediated mechanisms [185]. Other sources of lignans are foods rich in fibre, such as whole grains, legumes, several beverages (e.g., coffee and wine), oilseeds, fruits, and vegetables [183,186].

#### 3.2.2. Flavonoids

Flavonoids are one of the most common groups of polyphenolic compounds in the human diet. They are classified into chalcones, dihydrochalcones, aurones, flavones, flavonols, dihydroflavonoles, flavanones, flavanols, anthocyanidins, leucoanthocyanidins, proanthocyanins, bioflavonoids, and isoflavonoids [118,122]. In vitro and animal model studies show their ability to prevent diabetes and its subsequent complications. They have positive effects on carbohydrate and lipid metabolisms, and suppress hepatic enzyme activities [187,188]. They receive a lot of attention due to their structural diversity, abundance in nature, and strong pharmacological activity with low adverse reactions [141]. In this review, we characterize anthocyanins, epigallocatechin gallate, quercetin, naringin, rutin, and kaempferol, due to their excellent properties.

##### Anthocyanins

Anthocyanins are components of pigmented plant parts, that are flowers and fruits. The flavonoid or aglycone unit of anthocyanins is called anthocyanidin. The most frequent anthocyanins in plants are pelargonidin, cyanidin, peonidin, delphinidin, petunidin, and malvidin [189]. Generally, anthocyanins have significant antioxidant activities and are therefore used for the treatment of chronic and degenerative diseases such as diabetes, and cardiovascular and neurodegenerative disorders. Their antidiabetic properties are associated with lowering diastolic blood pressure and levels of triglycerides [190] and serum LDL cholesterol, as well as increasing HDL cholesterol concentrations [191]. Other beneficial effects include the prevention of free radical production and lipid peroxidation, improved insulin secretion and insulin resistance, and the inhibition of alpha-glucosidase activity [137]. However, data from studies with a large number of subjects and from research with isolated compounds, including anthocyanins, are still missing [190]. Significant sources of anthocyanins are berries (e.g., blackberries, bilberries, chokeberries, elderberries, cranberries, raspberries, blackcurrants, cherries, grapes, strawberries, coloured cabbages, and eggplants) [189].

##### Epigallocatechin Gallate

Epigallocatechin gallate is a type of catechin found mainly in green tea manufactured from *Camellia sinensis*. It contributes to weight loss by directly affecting adipose tissue. Epigallocatechin gallate can mimic insulin, increase the tyrosine phosphorylation of insulin receptors and the insulin receptor substrate, and downregulate the expression of enzyme phosphoenolpyruvate carboxykinase responsible for gluconeogenesis [138]. Moreover, it has antioxidant and anti-inflammatory features, and increases glucose-stimulated insulin secretion, as well as increasing the number and size of pancreatic islets [129,140,192]. On the other hand, the negative effects of epigallocatechin gallate on β-cells were revealed in the study by Yun et al. [193], where alloxan-induced diabetic rats showed an impaired islet cell mass loss and insulin immunoreactivity in β-cells. This conflict study points to the need for further research.

##### Quercetin

Quercetin (3,5,7-trihydroxy-2-(3,4-dihydroxyphenyl)-4Hchromen-4-one) is a flavonoid naturally occurring in plants and natural foods such as fruit and vegetables. It has many positive effects (e.g., anti-inflammatory, anti-oxidative, antihypertensive, anticancer, antiviral, neuroprotective, and hepatoprotective) [194]. It reduces the formation of reactive oxygen species (ROS), inhibits lipid peroxidation, increases plasma levels of adiponectin and HDL cholesterol. In addition, it has strong antidiabetic properties. It can enhance glucose uptake by a MAPK insulin-dependent mechanism and increase the phosphorylation of PI3K/Akt signalling pathways. This condition leads to the translocation of the glucose transporter 4 and downregulation of the activity of gluconeogenesis enzymes in the liver [141,142]. Quercetin also interacts with the PPARγ receptor [127]. Its consumption improves the action of β-cells [143] and proliferation, and inhibits alpha-glucosidase and alpha-amylase activities. Quercetin may improve diabetic bone disorder in patients with T2DM. It increases serum levels of calcium (Ca), vitamin D, and osteocalcin which improve bone mineralization. Its main disadvantage is the low bioavailability. Quercetin can be found in onions, apples, berries, many nuts, seeds, barks, flowers, tea, brassica vegetables, and leaves [141,195].

##### Naringin

Naringin (4′,5,7-trihydroxyflavanone 7-rhamnoglucoside) is a flavanone glycoside, present in citrus fruits. It has antioxidant, anti-inflammatory, anti-apoptotic, antiulcer, anti-osteoporotic, and anti-carcinogenic properties [196]. Moreover, the antihyperlipidaemic and antihyperglycaemic activities of naringin have also been revealed. It improves insulin signalling [144] and reduces blood glucose and cholesterol levels in experimental animals and patients with T2DM. The mechanism of its action is possible through the upregulation of PPARγ (it improves pancreatic β-cells cell dysfunction), and the upregulation of 5′ AMP-activated protein kinase (AMPK) in skeletal muscle cells (it increases glucose uptake). Furthermore, naringin can inhibit serum DPP-4 levels, enhance hepatic glycolysis and glycogen levels, and decrease hepatic gluconeogenesis. It can also improve mitochondrial dysfunction in the liver, and vascular dysfunction in obese Wistar rats [129,145,146]. The study by Liu et al. [197] discovered the potential of naringenin (part of naringin) in the prevention of bone damage by reduced overactivated bone resorption under T2DM conditions.

##### Rutin

Rutin (5,7,3′,4′-OH, 3-rutinose) is a plant-derived glycoside. It is a strong antioxidant that can scavenge free radicals and inhibit lipid peroxidation. It also has anti-inflammatory and anti-carcinogenic properties, and myocardial- and hepato-protecting activities [198,199,200]. Its antidiabetic and anti-inflammatory properties include lowering blood glucose levels, modulating insulin secretion, improving dyslipidaemia conditions, inhibiting AGEs formation, and positively influencing IRS-2/PI3K/Akt/GSK-3β signalling pathways [147]. Rutin may be found in fruits and fruit rinds, especially in citrus fruits, e.g., oranges, grapefruits, lemons, and limes. However, it can also occur in other food, such as buckwheat seeds [199], onions, apples, tea, and red wine [198].

##### Kaempferol

Kaempferol (3,5,7-trihydroxy-2-(4-hydroxyphenyl)-4*H*-1-benzopyran-4-one) is a flavonol with anti-inflammatory, antihypertensive, lipolytic, and anti-cancer properties [201]. In addition, it is an important regulator of lipid metabolism. It lowers plasma lipid levels, hepatic triglyceride and cholesterol levels, and reduces the accumulation of hepatic lipid droplets [202]. The antidiabetic activities of kaempferol are associated with the prevention of hyperglycaemia development, the suppression of hepatic gluconeogenesis through the reduction of pyruvate carboxylase activity [148]. Kaempferol may also improve insulin sensitivity by inhibiting pro-inflammatory cytokines, leading to reduced inflammatory responses and hepatic inflammatory lesions [149]. Kaempferol is presented in tea, cruciferous vegetables, grapefruit, *Gingko biloba*, and some edible berries [201].

#### 3.2.3. Plant Fruits, Vegetables and Other Products

In recent years, eco-friendly, bio-friendly, cost-effective plant-based medicines have come to the forefront of modern medicine thanks to more intensive research in the field of traditional medicine. There are several literature reviews by various authors focusing on antidiabetic plants and supplements [5]. In this review, selected plant fruits, vegetables and other products containing bioactive substances with demonstrable beneficial effects are listed below.

##### Garlic

Garlic (*Allium sativum*) is a flowering plant growing from a bulb. Its consumption has beneficial anticoagulant, antioxidant, antihyperlipidaemic, and antihypertensive effects. The antidiabetic active substances of garlic include allicin and hydrogen sulphide. Garlic supplementation promotes increased insulin secretion and sensitivity in animals [150,151]. On the contrary, studies demonstrating its hypoglycaemic impacts on humans are contradictory. The meta-analysis of Hou et al. [152] revealed a reduced postprandial blood glucose level in humans consuming garlic, while that of Emami et al. [151] revealed opposite findings. The potential side effects of consuming garlic include allergic contact dermatitis, allergic conjunctivitis, rhinitis, bloating, headache, dizziness, and profuse sweating [150].

##### Green Tea

Green tea is an unfermented tea derived from the plant *Camellia sinensis*. The active substances of green tea include catechins, especially epigallocatechin gallate, epigallocatechin, epicatechin gallate, and epicatechin [154]. The results indicate that the antidiabetic properties of green tea drinking are not clear. According to Sharma et al. [153], green tea consumption can increase insulin secretion, lower blood glucose levels, improve insulin resistance, and reduce diabetic complications. These assumptions are supported by the study of Liu et al. [154], who detected decreased glucose concentrations and increased insulin binding. Some studies have also demonstrated the ability of green tea to reduce blood pressure, LDL cholesterol, oxidative stress, and chronic inflammation [203]. In contrast, several studies have found no significant effect of drinking green tea on glucose control [204,205]. Green tea has been shown to reduce bone resorption markers and to positively affect bone turnover in patients with T2DM [206].

##### Blackcurrant

Blackcurrant (*Ribes nigrum* L.) is a medium-sized shrub grown for its edible fruits (berries). The berries contain high levels of anthocyanins, principally delphinidin 3-rutinoside, but also cyanidin-3-rutinoside, delphinidin-3-glucoside, and cyanidin-3-glucoside, as well as condensed tannins (proanthocyanidins), oligomeric, and polymeric chains of flavan-3-ols. Various blackcurrant extracts and beverages can decrease blood glucose levels, enhance glucose tolerance in obese mice and rats, and reduce postprandial blood glucose concentrations in humans with T2DM. Their action is mediated by the inhibition of alpha-glucosidase and alpha-amylase activities. Blackcurrants have a similar effect to alpha-glucosidase inhibitors; they delay carbohydrate digestion and inhibit the rate of glucose absorption in the intestine. They can also potentiate the effect of acarbose [43,155,156]. Moreover, blackcurrants have a phytoestrogenic activity and can positively influence bone structure [207], which could also indicate its beneficial effect on diabetic bone damage.

##### Rowanberry

Rowanberry (*Sorbus aucuparia*) is grown as an ornamental plant as well as for fruit. The active substances of the berries include ascorbic acid, carotenoids, flavonoids, anthocyanins, and phenolic acids with demonstrable antihyperlipidaemic, anti-inflammatory, and antidiabetic effects [208]. Like blackcurrants, rowanberries can also inhibit alpha-glucosidase activity, delay the absorption of carbohydrates in the small intestine, and potentiate the impact of acarbose [43,157]. The study by Grussu et al. [158] demonstrated their ability to inhibit alpha-amylase in vitro. Because acarbose has adverse side effects, supplementation with rowanberries may be an equivalent compensation, but further investigation is needed.

##### Bilberry

The bilberry or European blueberry (*Vaccinium myrtillus* L.) is a perennial shrub grown mainly for fruit. The active therapeutic compounds found in the berries and leaves contain anthocyanins, quercetins, catechins, tannins, iridoids, phenolic acids, vitamins, and sugars. A higher content of flavonoids was recorded in the leaves compared to berries. Bilberries have strong antioxidant and anti-inflammatory properties; they can be used to treat vision disorders, vascular diseases, and T2DM [160,209,210]. According to Güder et al. [159], consumption of bilberries can reduce alpha-amylase and alpha-glucosidase activities due to their high content of anthocyanins. Bilberry extract significantly decreases hyperglycaemia, and has a positive effect on weight gain in ZDF rats. Decreased glucose levels and weight loss were also determined in streptozotocin (STZ)-induced diabetic rats supplemented with bilberry leaf extract [160].

##### Strawberry

The strawberry (*Fragaria vesca*) is a plant containing high amounts of vitamin C, folate, and phenolic constituents. It belongs to a group of foods rich in anthocyanins. The antitumor, antioxidant and antidiabetic effects of the fruit have been recorded in several studies. Strawberries have a beneficial impact on ageing, obesity, cardiovascular disease, and metabolic syndrome [128,211]. They can lower postprandial glucose concentrations and improve insulin sensitivity [161,162]. These effects are achieved thanks to the anthocyanin pelargonidin-3-O-rutinoside, which is a proven alpha-glucosidase inhibitor [128,212].

##### Cornelian Cherry

The cornelian cherry (*Cornus mas*) is a medicinal plant cultivated as an ornamental plant. Its fruits contain high levels of anthocyanins (10–15 times more than other fruits) [213,214], quercetin, aromadendrin [215], vitamins (e.g., C, E, and B), polyphenols, minerals (e.g., iron (Fe), Ca, potassium (K), sodium (Na), Zn, and manganese (Mn)), and antioxidants [216]. These compounds have anti-inflammatory, anticancer, antidiabetic properties and positively affect cardiovascular and nervous systems [217]. Cornelian cherry fruits can lower LDL and HDL cholesterols [215], triglycerides, total cholesterol, and alkaline phosphatase activity [218]; decreased blood glucose levels have also been reported [163]. According to Omelka et al. [218], the beneficial impact of Cornelian cherry pulp on T2DM-impaired bone quality was determined in ZDF rats, indicating its potential to alleviate T2DM-reduced bone health. Nevertheless, the expected synergistic effect of cornelian cherries and bee bread on diabetic bone disorder has not been confirmed in the appropriate animal model [219].

##### Olive Oil

Olive oil is a liquid fat extracted from the fruit of the olive tree (*Olea europaea*). Its biological activity is attributed to high concentrations of monounsaturated fatty acid, phenolic compounds [220], and oleic acids. Its extra-virgin form is used as a herbal remedy in the treatment of neurodegenerative diseases, diabetes mellitus, obesity, atherosclerosis, cardiovascular disorder, metabolic syndrome, and certain forms of cancer. Generally, olive oil can improve postprandial lipidaemia and glucose homeostasis in humans. It is also used as a low-fat diet in the nutritional therapy of T2DM [221]. The study by Rajput et al. [220] showed its beneficial impact on the lipid profile of diabetic rats (increased HDL cholesterol levels and decreased total cholesterol, triglyceride, and LDL cholesterol levels). According to Alkhatib et al. [164], its consumption acts preventively against inflammation and oxidative stress in pancreatic β-cells, improves β-cells capacity, insulin resistance and adipocyte differentiation. Moreover, it can have a protective effect against diabetic bone damage due to elevated serum osteocalcin levels in elderly patients [222]. Phenolic compounds in extra virgin olive oil enhance the proliferative capacity and differentiation of osteoblasts and inhibit osteoclastogenesis [223].

##### Sesame Oil

Sesame oil is an edible vegetable oil obtained from the seeds of sesame (*Sesamum indicum*). It contains monounsaturated fatty acids and polyunsaturated fatty acids, especially linoleic acid, and oleic acid. In addition, it is a rich source of vitamin E, lignan sesamin, and also contains sesamol, episesamin, and sesamolin. Sesame oil has antioxidant, antidiabetic, and antihypertensive properties [165,224,225]. The study by Ramesh et al. [165] reported a significant reduction in blood glucose levels in diabetic rats following sesame oil administration. According to Alam et al. [43], its impacts were due to the ability to inhibit alpha-glucosidase activity and to possess an insulin-like effect. In patients with T2DM, sesame oil consumption lowers levels of total cholesterol and LDL cholesterol, and increases HDL cholesterol concentration [224].

##### Carrot

The carrot (*Daucus carota* Linn.) is a root vegetable belonging to the *Apiaceae* family. In general, Se, vitamins (e.g., A, B, C, and E), flavonoids, and glutathione are considered to be biologically active substances in carrots. The roots have anti-inflammatory and antioxidant properties [226,227]. Moreover, they are rich in the number of fibres associated with cholesterol metabolism, as well as carotenoids consistent with antioxidant capacity [228]. According to Khaki et al. [226], simultaneous supplementation with carrot seeds and ginger reduced diabetic nephropathy in STZ-induced diabetic rats. The consumption of carrot seeds reduced serum levels of total cholesterol, triglycerides, and LDL cholesterol in STZ-induced diabetic rats [227]. The study by Kumar et al. [166] pointed to the antidiabetic, haematinic and anti-cholesterolemic impacts of carrot juice supplementation in alloxan-induced diabetic rats.

### 3.3. Combination Therapy

Because T2DM is a progressive disease characterized by escalating hyperglycaemia, its treatment requires the use of higher doses or combination therapy to maintain glycaemic control [48,81]. The first choice of therapy is usually metformin, which is replaced by sulphonylureas in case of metformin contraindications and/or unsuccessful monotherapy. Thiazolidinediones are used when certain contraindications to metformin have been reported. They can also be used simultaneously with metformin. Alpha-glucosidase inhibitors are recommended as an alternative glucose-lowering therapy in patients who are unable to use other pharmaceutical drugs [229,230]. Meglitinides can be administered separetely or in combination with metformin [78]. DPP-4 inhibitors are used both as monotherapy and simultaneously with metformin, sulphonylureas, or thiazolidinediones [231]. Prescribing an appropriate combination of medications requires knowledge of many classes of drugs, information on individual agents, and knowledge of important differences between drugs in each class. What is particularly promising are the newest classes of drugs, based on GLP-1R agonists and SGLT2 inhibitors, which currently have a fundamental role in cardiovascular and renal risk reduction in patients with T2DM. Recent studies have shown that even combination therapy with these two classes may produce additive cardiovascular and renoprotective benefits [232,233].

At an advanced stage of the disease, treatment of T2DM with only pharmaceutical agents may not be sufficient. The combination therapy of synthetic drugs and natural compounds provides a way to make the treatment more effective. Moreover, synergistic interactions can be helpful in reducing the dose of antidiabetic drugs while minimizing various adverse effects associated with their use [43]. Possible therapeutic combinations of pharmaceutical and natural products are mentioned below. The known synergistic effects and mechanisms of action of all combination therapies are listed in Table 2.

#### 3.3.1. Biguanides with Natural Products

The antidiabetic synergistic effects of biguanides with certain natural substances have been determined. Several studies suggest a synergistic impact of resveratrol and metformin [234]. Furthermore, decreased blood glucose levels have been observed in STZ-induced diabetic rats receiving garlic extract in combination with metformin [235]. The study by Kannappan and Anuradha [243] revealed enhanced insulin sensitivity in a rat model after supplementation with polyphenolic extract or quercetin, and this effect was similar to that of metformin.

#### 3.3.2. Thiazolidinediones with Natural Products

The combination therapy with thiazolidinediones and selected natural compounds can also ameliorate T2DM-related complications. Resveratrol, curcumin, and quercetin are able to interact with the PPARγ receptor, as well as pioglitazone and other thiazolidinediones [127], suggesting their ability to increase the efficacy of these pharmaceuticals. This fact was confirmed by the study of Prasad [236], who revealed a beneficial impact of simultaneous administration with curcumin and pioglitazone on the pharmacokinetics and pharmacodynamic in alloxan-induced diabetic rats.

#### 3.3.3. Sulphonylureas with Natural Products

It is known that hyperglycaemic conditions in patients with T2DM or STZ-induced diabetic rats can be achieved by combining sesame oil or garlic extract with glibenclamide, respectively [225,244]. This combination therapy reduced the total cholesterol, LDL cholesterol, and triglyceride levels, and increased HDL cholesterol concentration in diabetic patients [225]. In addition, garlic extract in combination with another sulphonylurea, gliclazide, significantly decreased blood glucose level in STZ-induced diabetic rats [237]. Glimepiride (another member of the sulphonylurea group) with curcumin had protective effects against diabetic alterations associated with selected biochemical parameters and total antioxidant status in diabetic rats [238].

#### 3.3.4. Meglitinides with Natural Products

Only one study examined the impact of the co-administration of meglitinides and natural products on T2DM conditions. Simultaneous supplementation with curcumin and repaglinide decreased total cholesterol levels, reduced ROS and lipid peroxidation, and enhanced total proteins and serum insulin levels in STZ-induced diabetic rats [239].

#### 3.3.5. Alpha-Glucosidase Inhibitors with Natural Products

Many natural products were associated with alpha-glucosidase inhibitor activity. Some polyphenols (e.g., epigallocatechin gallate or quercetagetin) had comparable effects to acarbose [245]. The co-administration of acarbose with green tea extract enhanced its effect on inhibiting alpha-amylase and alpha-glucosidase activities [240]. On the other hand, the combination of green tea with acarbose was ineffective in the study by Villa-Rodriguez et al. [246] The activity of alpha-glucosidase may also be reduced by anthocyanin-rich combinations of blackcurrant or rowanberry [43,157].

#### 3.3.6. Incretin-Based Therapies with Natural Products

Some natural compounds influenced incretins such as GLP-1R agonists and DPP-4 inhibitors. The synergistic effect of epigallocatechin gallate in combination with exendin-4 (GLP-1R agonist) improved glycaemic control, insulin release, insulin sensitivity, and dyslipidaemia in diabetic mice [241]. Anthocyanins are well-known DPP-4 inhibitors; black carrot extract is even more effective than acarbose and/or vildagliptin [190]. The results of the study by González-Abuín et al. [247] showed the same effectivity of anthocyanin-rich extract and vildagliptin, when administered individually, to inhibit DPP-4 activity in Wistar rats, but their research did not include a group simultaneously supplemented with both agents.

#### 3.3.7. SGLT2 Inhibitors with Natural Products

Only one study investigated the effect of the co-administration of dapagliflozin and resveratrol on T2DM conditions using ob/ob mice and HK-2 cells (human renal proximal tubule cells). After resveratrol treatment, dapagliflozin-induced renal glucose production and gluconeogenesis were alleviated through activating the PI3K/Akt pathway and supressing FoxO1 activation. This simultaneous supplementation had the potential to improve glucose-lowering effects for SGLT2 inhibitors in T2DM therapy [242].

## 4. Conclusions

The currently recommended treatment for T2DM involves a combination of effective lifestyle changes and the use of pharmaceutical therapy. The primary care in T2DM is pharmacological, especially considering the beneficial effects of new antidiabetic classes of drugs (GLP-1R agonists and SGLT2 inhibitors) on the primary and secondary prevention of cardiovascular and kidney diseases. These medications can be used in the first instance together with metformin in T2DM patients at risk.

The long-term administration of pharmaceutical drugs can cause many negative side effects. For this reason, researchers are encouraged to study natural health products that offer a therapeutic alternative to primary pharmacological therapy through various mechanisms. They can be recommended as food supplements to prevent and/or alleviate complications associated with T2DM. The use of natural substances may be indicated not only in the pre-diabetic stage and in the early stage of T2DM, but also in the advanced stage of this disease.

There are still many questions and limitations regarding the application of natural compounds as potential drugs for the treatment of T2DM. Many of those characterized in this review have hypoglycaemic effects. When used at higher doses, patients may develop hypoglycaemia. Therefore, further studies are needed which focus on dosing, standard preparation, and the detailed characterization of these substances. Moreover, the long-term side effects and possible toxicity of natural compounds needs to be monitored. To date, some herbal toxicities have been identified, of which hepatotoxicity is the most commonly reported toxic effect. Plants generally produce various toxic compounds as secondary metabolites which may limit the use of active pharmacological ingredients. Some of these herbs are produced under environmentally unsuitable conditions (especially in developing countries) using potentially toxic chemicals [248]. In addition, the beneficial effects of some natural substances have been revealed in in vitro studies but have not been validated in in vivo studies. Most natural compounds with antidiabetic properties have not been clinically tested, and if so, clinical studies often include a limited number of individuals or do not consider the different characteristics of patients with T2DM. Further studies are also needed to reveal the cellular and molecular mechanisms of action of natural compounds, as well as their combinations with current pharmaceutical drugs. In particular, the potential chemical interactions of natural substances with other synthetic products should be carefully considered. For example, plants containing caffeine can interfere with sedative and adrenergic drugs, and garlic can interact with anticoagulants. There may be a potential interaction between dual-acting medicinal products and drugs. An example is olive leaves where their use to improve diabetes can lead to a hypotensive crisis [249]. In addition, various herbal products have antidiabetic effects potentially mediated by the modulation of gut microbiota, which is another promising area of the research [250].

In this review, we focused on the detailed characteristics of available pharmaceutical drugs and selected natural products, which were used to treat T2DM, as well as their combination therapies. The effects and mechanisms of their action in alleviating T2DM-related complications, including diabetic bone disease, have also been described. We suggested that the natural substances mentioned in this manuscript were beneficial in the treatment of T2DM and have considerable potential to be used as perspective drugs or dietary supplements. However, more high-quality pre-clinical studies and clinical trials are needed to provide evidence of their dosage, long-term side effects, possible toxicity, mechanisms of action, and interactions.

## Figures and Tables

**Table 1:** List of all natural products reported in this review.

Natural Products	Models Used	Known Effects and Mechanisms of Action	References
**NON-FLAVONOID POLYPHENOLS**			
resveratrol	patients with T2DM (9 randomized controlled trials, *n* = 283)	improves glucose control and insulin sensitivity	[124]
	diet-induced obese mice and rats, ZDF rats	enhances insulin sensitivity by activation of deacetylases sirtuins 1–7, protects β-cells from oxidative stress, interacts with PPARγ receptor	[124,125,126,127]
	in vitro (different cell lines)	improves insulin signalling and inhibits oxidative stress	[125,126]
curcumin	obese patients (randomized, cross-over study, *n* = 16)	reduces postprandial glycaemia	[128]
	C57BL/6J HFD-induced obese mice	improves insulin sensitivity	[128]
	STZ-induced rats	supports pancreatic cell viability by inhibition of lipid peroxidation, NF-κB activation and reduction of inflammatory cytokine levels	[129]
	in vitro (enzyme inhibition assay)	inhibits alpha-amylase and alpha-glucosidase activities	[43]
tannins	in vitro (enzyme inhibition assay)	inhibit alpha-glucosidase activity	[130]
	in vitro (3T3-L1 adipose cells)	improve insulin sensitivity	[130]
	in vivo and in vitro models	have insulin-like effect, stimulate glucose transport, reduce formation and accumulation of AGEs	[131,132]
lignans	patients with pre-diabetes (randomized cross-over trial, *n* = 25)	decrease blood glucose levels, delay postprandial glucose absorption, reduce inflammation and oxidative stress	[133]
	diet-induced obese mice	reduce blood glucose and insulin levels, improve oral glucose tolerance, insulin signalling and insulin sensitivity	[134]
	STZ-induced rats, ZDF rats	delay onset of diabetes	[135,136]
**FLAVONOIDS**			
anthocyanins	several cross-over trials on humans	prevent free radical production and lipid peroxidation, increase insulin secretion, improve insulin resistance, inhibit alpha-glucosidase activity	[137]
epigallocatechin gallate	db/db mice, ZDF rats	affects gluconeogenesis by downregulation of enzyme phosphoenolpyruvate carboxykinase, increases tyrosine phosphorylation of insulin receptors	[138]
	db/db mice, alloxan-induced diabetic mice	has antioxidant and anti-inflammatory abilities, enhances glucose-stimulated insulin secretion	[129,139,140]
	db/db mice	increases number and size of pancreatic islets	[129]
quercetin	in vivo and in vitro models	enhances glucose uptake by a MAPK insulin-dependent mechanism, increases phosphorylation of PI3K/Akt signalling pathways, interacts with PPARγ receptor, inhibits alpha-glucosidase and alpha-amylase activities	[127,141,142]
	STZ- induced rats	improves β-cells action	[143]
naringin	fructose-fed rats	improves insulin signalling	[144]
	high-carbohydrate + high fat-fed rats	improves mitochondrial dysfunction in the liver	[145]
	high fat-fed rats	reduces blood glucose and cholesterol levels by upregulation of PPARγ	[146]
	in vitro (rat skeletal L6 myoblast cell line)	upregulates of 5′ AMPK in skeletal muscle cells	[146]
	in vivo and in vitro models	inhibits serum DPP-4 levels	[129]
	db/db mice	enhances hepatic glycolysis and glycogen concentration, reduces hepatic gluconeogenesis	[129]
rutin	db/db mice	reduces blood glucose level, modulates insulin secretion, inhibits AGEs formation, positively affects IRS-2/PI3K/Akt/GSK-3β signalling pathway	[147]
kaempferol	diet-induced obese mice	prevents hyperglycaemia development, suppresses hepatic gluconeogenesis by reducing pyruvate carboxylase activity	[148]
	STZ-induced + high-fat diet rats	improves insulin sensitivity by inhibiting pro-inflammatory cytokines, leading to reduced inflammatory responses and hepatic inflammatory lesions	[149]
**PLANT FRUITS, VEGETABLES AND OTHER PRODUCTS**			
garlic	in vivo and in vitro models	increases insulin secretion and sensitivity	[150,151]
	obese patients with T2DM (open-label, prospective, comparative trial, *n* = 60)	reduces postprandial blood glucose level	[152]
green tea	in vivo and in vitro models	increases insulin secretion, lowers blood glucose levels, improves insulin resistance, reduces diabetic complications	[153]
	in vitro (adipocytes isolated from rats)	reduces concentration of glucose and increases insulin binding	[154]
blackcurrant	patients (randomized, double-blind, cross-over trial, *n* = 23)	reduces postprandial blood glucose level	[155]
	diet-induced obese mice, high-fructose diet rats	decreases blood glucose levels, enhances glucose tolerance	[156]
	in vivo and in vitro models	delays carbohydrate digestion, inhibits glucose absorption in the intestine	[155]
	in vitro (enzyme inhibition assay)	inhibits alpha-glucosidase and alpha-amylase activities, increases the effect of acarbose	[145,157]
rowanberry	in vitro (enzyme inhibition assay)	inhibits alpha-glucosidase and alpha-amylase activities, increases the effect of acarbose	[157]
	in vitro (enzyme inhibition assay)	inhibits alpha-amylase	[158]
bilberry	in vitro (enzyme inhibition assay)	inhibits alpha-amylase and alpha-glucosidase activities	[159]
	ZDF rats	decreases hyperglycaemia, positively affects body weight gain	[160]
	STZ-induced rats	reduces glucose level and weight loss	[160]
strawberry	obese patients (single-blinded, cross-over trial, *n* = 14)	lowers postprandial glucose concentrations, improves insulin sensitivity	[161]
	obese patients with insulin resistance (randomized, single-blinded, diet-controlled crossover trial, *n* = 21)	decreases postprandial glucose concentrations, improves insulin sensitivity	[162]
cornelian cherry	ZDF rats	reduces blood glucose level	[163]
olive oil	in vivo and in vitro models	acts preventively against inflammation and oxidative stress in pancreatic β-cells, improves β-cells capacity, insulin resistance and adipocyte differentiation	[164]
sesame oil	STZ-induced rats	reduces blood glucose level	[165]
	in vitro (3T3-L1 adipose cells)	inhibits alpha-glucosidase activity, possesses insulin-like effect	[43]
carrot	alloxan-induced diabetic rats	has antidiabetic, haematinic and anti-cholesterolemic impacts	[166]

STZ: streptozotocin; ZDF: Zucker diabetic fatty; T2DM: type 2 diabetes mellitus; PPAR-γ: peroxisome proliferator-activated receptor-γ; NF-κB: nuclear factor kappa-light-chain-enhancer of activated B cells; AGEs: advanced glycation end products; MAPK: mitogen-activated protein kinase; DPP-4: dipeptidyl peptidase 4; 5′ AMPK: 5′ adenosine monophosphate-activated protein kinase; PI3K/Akt: phosphatidylinositol-3-kinase/protein kinase B; IRS-2/PI3K/Akt/GSK-3β: Insulin Receptor Substrate 2/phosphatidylinositol-3-kinase/protein kinase B/glycogen synthase kinase 3β; *n*: number of individuals.

**Table 2:** List of all combination therapies reported in this review.

Combination Therapy	Models Used	Known Synergistic Effects and Mechanisms of Action	References
**BIGUANIDES WITH NATURAL PRODUCTS**			
metformin + resveratrol	in vivo and in vitro models	enhances hyperglycaemia, dyslipidaemia, insulin resistance, pro-inflammatory response, and lipid peroxidation	[234]
metformin + garlic extract	STZ-induced rats	reduces blood glucose levels	[235]
**THIAZOLIDINEDIONES WITH NATURAL PRODUCTS**			
pioglitazone + curcumin	alloxan-induced diabetic rats	has beneficial impact on the pharmacokinetics and pharmacodynamics	[236]
**SULPHONYLUREAS WITH NATURAL PRODUCTS**			
glibenclamide + sesame oil	patients with T2DM (open label study, *n* = 60)	has anti-hyperglycaemic, anti-hypercholesterolemic effects, antioxidant activity	[225]
glibenclamide + garlic extract	STZ-induced rats	has hypoglycaemic effect, increases body weight	[47]
gliclazide + garlic extract	STZ-induced rats	reduces blood glucose level	[237]
glimepiride + curcumin	STZ-induced rats	has protective effects against diabetic alterations associated with selected biochemical parameters and total antioxidant status	[238]
**MEGLITINIDES WITH NATURAL PRODUCTS**			
repaglinide + curcumin	STZ-induced rats	reduces ROS and lipid peroxidation, enhances total proteins and serum insulin levels	[239]
**ALPHA-GLUCOSIDASE INHIBITORS WITH NATURAL PRODUCTS**			
acarbose + green tea extract	in vitro (enzyme inhibition assay)	inhibits alpha-amylase and alpha-glucosidase activities	[240]
**INCRETIN-BASED THERAPIES WITH NATURAL PRODUCTS**			
exendin-4 + epigallocatechin gallate	high-fat diabetic mice	improves glycaemic control, insulin release, insulin sensitivity and dyslipidaemia	[241]
**SGLT2 INHIBITORS WITH NATURAL PRODUCTS**			
dapagliflozin + resveratrol	ob/ob mice + HK-2 cells	alleviates dapagliflozin-induced renal glucose production and gluconeogenesis through activating the PI3K/Akt pathway and supressing FoxO1 activation	[242]

STZ: streptozotocin; T2DM: type 2 diabetes mellitus; ROS: reactive oxygen species; *n*: number of individuals; FoxO1: Forkhead Box O1.

## Data Availability

Data sharing not applicable.
